# Innovation in Orthotics: Development of Technical Textiles from Bamboo Cellulose

**DOI:** 10.3390/polym18060669

**Published:** 2026-03-10

**Authors:** Willam Ricardo Esparza, Wilson A. Herrera-Villarreal, Lenin Omar Lara Castro

**Affiliations:** Facultad de Ingeniería en Ciencias Aplicadas, Universidad Técnica del Norte, Ibarra 100150, Ecuador

**Keywords:** bamboo cellulose, medical orthoses, polymer composites, sustainable biomaterials, mechanical properties

## Abstract

This study evaluated the relevance of using bamboo cellulose (BC) compounded with resin (R) for the manufacture of medical orthoses (BCO). A 22-factorial screening experimental design was used, with two experimental factors and six response variables. Three polymer composites (PC) were prepared: S1 (BC 40%, R 60%), S2 (BC 30%, R 70%), and S3 (BC 20%, R 80%), which were molded under a pressure of 10.5 kg in 25 × 5 cm male-female dies, with an internal space of 2 mm, at 20 °C for 24 h. The mechanical properties evaluated included tensile strength (RTRAC), ball penetration resistance (RPEBOL), puncture resistance (RPUNZ), and their corresponding extensions (ETRAC, EPEBOL, and EPUNZ). Mass, tensile strength, elongation, punching resistance, and penetration were determined in accordance with ISO 3801, ISO 9073-3, EN 388, and ASTM D3787 standards. Statistical analysis was performed using Statgraphics Centurion and Past 4.13 software. The results showed that increasing the resin content and decreasing the bamboo cellulose significantly improved the mechanical performance of the material. The S3 samples (BC 20%, R 80%) had the highest mechanical strength values, with a tensile strength of (1049.34 ± 85.57 N; n = 5), representing an increase of 398.60% over the base formulation. Likewise, increases of 92.25% in puncture resistance (24.12 ± 29.91 N; n = 5) and 196% in ball penetration resistance (323.98 ± 1.39 N; n = 5) were recorded. Tensile elongation showed an increase of 228% (7.55 ± 5.01%; n = 5). In the S2 samples (BC 30%, R 70%), the greatest increase was observed in the puncture elongation, with a value of 16.33 ± 1.25 mm (n = 5), corresponding to an increase of 59.78%. Meanwhile, the S1 samples (BC 40%, R 60%) exhibited the highest ball penetration extension value (34.07 ± 1.61 mm; n = 5), while the S2 and S3 formulations recorded decreases of 2.11% and 2.23%, respectively. Additionally, thickness, weight, and density showed a strong correlation with each other (*p* > 0.05). Overall, the results indicate that the combination of bamboo cellulose and epoxy resin is a sustainable and effective alternative for the development of medical orthoses, due to the significant improvement in their mechanical properties, which supports their application in orthotic devices based on sustainable biomaterials.

## 1. Introduction

Innovation in the field of external orthoses has seen significant advances in recent years, driven by the need to improve the quality of life for people with physical disabilities. The most important properties they must meet are mechanical strength, balanced rigidity and flexibility, lightness, dimensional stability, malleability, and processability. Traditionally, these were manufactured using plaster molding techniques [1]; however, these materials have high rigidity and structural weight, so they do not meet the required properties. In contrast, external orthoses made from polymeric materials such as carbon composites have been widely used in medical devices, including orthoses and prostheses [2]. These materials contain epoxy resins, which depend mainly on non-renewable petroleum resources, commonly of the diglycidyl ether bisphenol A (DGEBA) epoxy monomer type [3]. Currently, these applications have been extended to the manufacture of solid external orthoses by fused deposition modeling, using materials such as polylactic acid (PLA) and carbon fiber reinforced PLA (PLA-C) by FDM 3D printing [4]. As therapeutic offloading devices, including other external orthoses, these systems may be more effective than standard offloading devices [5], as they improve rotation and flexion, help to increase skeletal load, and prevent fractures [6]. This has been reported by studies where biomechanical evaluations indicate that the flexor tendons in the finger joints generate forces in the range of 8.15 N for full flexion [7]. Similarly, in another study, using 3D printing with modified polyethylene terephthalate glycol (PETG), a breaking strength of 45.17 MPa and an extension of 22.19 kgf were obtained, indicating that the material can withstand high levels of stress before breaking [8]. Meanwhile, another study determined that PLA (polylactic acid) is the most suitable material for orthopedic design. Strength, flexibility, weight, cost, and ease of manufacture were considered, and the results revealed that the minimum thickness required for PLA orthoses is 3 mm. The results revealed that the maximum stress recorded is 24.32 MPa, ensuring that it meets the criteria for withstanding stress and deformation [9]. Another study conducted using a vacuum molding technique based on two types of materials as composite reinforcement was based on eight layers of Perlon (acrylic), while the second used eight layers of fiberglass. The findings revealed that the strength performance was 42.897 MPa; the tensile strength for Perlon was 42.993 MPa; and the elongation at break was 1.138 mm. In contrast, fiberglass exhibited a tensile strength, strength performance, and elongation at break of 224 MPa, 170 MPa, and 2.17 mm, respectively [10]. In addition, other authors explored filaments composed of thermoplastic polyurethane (TPU)/polylactic acid (PLA) for 3D printing. The polymer compositions considered were TPU/PLA: 0%/100% (TPO), 25%/75% (TP25), and 50%/50% (TP50). Compared to pure PLA, the TP25 samples exhibited almost equal tensile strength; however, their greater elongation at break indicates that TP25 is more suitable as a material for orthoses. However, a further increase in TPU to 50% resulted in a marked decrease in tensile strength [11]. Another study noted that the composites incorporated abaca fiber (AF), epoxy (EP), and activated carbon particles (ACP). The results showed that the addition of 5 vol% ACP to AF/EP led to an optimum tensile strength of 42.50 MPa, slightly lower than AF/EP (43.00 MPa), and an optimum flexural strength of 62.10 MPa, slightly higher than AF/EP (60.50 MPa). Furthermore, the addition of 20% ACP to EP resulted in a tensile strength of 31.93 MPa, higher than the previous result for ACPs/EP (26.34 MPa) [12]. Other sources manufactured using fused deposition modeling (FDM) under different printing conditions, with a layer thickness of 0.1 mm, found a tensile strength close to 28 MPa for polypropylene (PP) samples [13]. Likewise, this study investigates a multilayer composite (MLC) that integrates a 3D-printed (3DP) core with technical fiber reinforcement and epoxy resin. The material was manufactured with a flat PA12 3DP core using powder bed fusion, applying unidirectional glass fibers using customized fiber placement (TFP) and wrapping it in a biaxial carbon fiber braided sleeve. The MLC exhibited a tensile strength of approximately 300 MPa [14]. Similar observations were presented in experimental findings based on Orthocryl, reinforced with multi-walled carbon nanotubes (MWCNT) and polylactic acid (PLA). The incorporation of 0.5% MWCNT into Orthocryl was shown to significantly improve its mechanical properties, with a 12.5% increase in tensile strength (from 52.79 to 59.4 MPa) [15]. Similarly, in 3D printing using topological optimization and finite element analysis (FEA), printed samples were developed, obtaining tensile strength results of 53 MPa under a load of 100 N. In addition, stresses of 0.017–0.186 MPa, displacements of 0.0155–0.0361 mm, and deformations of 0.00044–0.00184 were predicted, with weight reduction and low stress (0.1479 MPa) and displacement (0.0208 mm) values [16]. With regard to degradation, the amorphous magnesium phosphate (AMP) composite with polyetheretherketone (PEEK) exhibits controlled degradation kinetics, with tensile strength progressively decreasing from 120 to 70 MPa over a period of 28 days due to hydrolytic degradation [17]. Among the various reinforcement techniques, fiber-reinforced polymers (carbon, glass, aramid, Kevlar, among others) have proven beneficial in improving the overall performance of aged and damaged structural elements [18]. Similarly, previous studies have reported the contribution of cellulose nanocrystal (CNC) mixtures in epoxy matrices, prepared by dispersing different concentrations of CNC (0.125, 0.25, and 0.5% by weight), achieving an improvement in fracture toughness [19]. Likewise, an improvement in the mechanical properties of epoxy resins has been reported through their modification with citric acid combined with cellulose (CAC) [20]. Other studies indicate that the addition of phenolic resins in epoxy matrices can improve the mechanical performance of epoxy-based resins and composites at room temperature [21]. In this context, it is necessary to search for new biodegradable materials that perform well while maintaining their functional properties [22]. In recent years, much research has been conducted in the search for sustainable and biodegradable material options. Among the alternatives proposed, bamboo cellulose stands out as a sustainable and economical biopolymer with low environmental impact and exceptional properties [23]. Bamboo grows rapidly, requires few resources, and offers strength, biodegradability, and therapeutic properties [24], allowing it to be used in the manufacture of orthopedic devices [25]. In addition, it is considered a promising biomass resource for addressing the energy crisis and climate change [26,27], with bamboo culm being a plant matrix with great potential due to its high content of low-crystallinity cellulose [28]. Previous studies have shown that both cane and bamboo are viable alternative materials for the manufacture of orthopedic devices, prosthetics, and rehabilitation devices [29]. Likewise, research has shown significant structural differences in bamboo fibers and cell walls at the nanometric scale [30]. Bamboo cellulose can be obtained using the hydrothermal technique, employing a treatment with 5% NaOH and 4% H_2_O_2_, achieving yields of around 32.56% [31]. This process allows the formation of a renewable natural hydrophilic polymer, generated after regeneration or mercerization processes [32]. On the other hand, bamboo cellulose extracted by the Kraft process in an alkaline medium consists of natural fibers combined with biopolymers, resulting in environmentally friendly compounds that are recyclable or biodegradable by nature [33]. To obtain nanocellulose, bamboo fibers can be treated with high-temperature and high-pressure steam, followed by a disintegration process until a nanometric scale is reached [34,35]. In this context, various authors have developed multiple applications based on bamboo cellulose, such as the manufacture of cellulose films obtained from mixtures of micro- and nanomaterials, through the rapid succinylation of bamboo cellulosic materials at room temperature, with reaction times of approximately 10 min [36]. Likewise, polylactic acid (PLA) composites reinforced with cellulose nanofibers have been prepared. These characteristics make bamboo cellulose a promising option for medical applications. Its incorporation into the manufacture of orthoses represents an innovation that not only improves the functionality and comfort of these devices but also promotes sustainability in the healthcare industry, given that it is an abundant and sustainable resource in tropical and subtropical regions, characterized by its rapid growth, short rotation, and greater mechanical resistance compared to other species [37]. These acquired properties, together with the technical development achieved, are fundamental for its application in the manufacture of lightweight, flexible, and clinically durable orthoses from bamboo cellulose [38]. To this end, the implementation of orthoses made from biodegradable materials based on bamboo cellulose is proposed, taking advantage of its interfacial properties through physical and chemical processes. The production of ecological and economical fibers from plant sources and waste, as well as the development of ecological, thermosetting, and thermoplastic composites, has high potential and multiple applications [39,40]. Unlike previous studies, in which research has focused mainly on the manufacture of composite materials using PLA, 3D printing, and carbon fiber reinforcements with cellulose-based polymer matrices, this study addresses the application of bamboo cellulose as a reinforcing material. The cellulose used was obtained and characterized previously in an earlier study. In addition, the composite material was formed using a traditional, manual method based on the creation of male and female molds and the application of controlled pressure of 10 kgf. Its mechanical properties were evaluated through tensile, punch, and ball penetration tests in order to determine its suitability for use in the manufacture of external orthoses. This represents a distinctive methodological contribution compared to the conventional approaches reported in the literature.

## 2. Materials and Methods

### 2.1. Materials

In this study, bamboo cellulose (BC) was extracted from *Guadua angustifolia* (subfamily Bambusoideae) from the parish of Lita, located in the northwest of the province of Imbabura, approximately 90 km from the city of Ibarra, Ecuador, at an altitude of 250 m above sea level. The process used to obtain BC was alkaline, using sodium hydroxide flakes at a concentration of 50% relative to the weight of the crushed bamboo, and drinking water in a bath ratio of 1:10. The treatment was carried out at a temperature of 90 °C for 6 h [41]. The CB obtained had a powdery morphology and a beige coloration previously characterized in an earlier study, with a pulverized particle size of 0.05 mm [42]. The molecular weight of bamboo cellulose was not determined experimentally in this study. However, according to values reported in the literature for bamboo celluloses subjected to similar grinding and physical treatment processes, the average molecular weight (Mw) typically ranges between 1.3 × 10^5^ and 3.2 × 10^5^ g/mol, with a degree of polymerization (DP) of bamboo greater than that of dicotyledonous woods (a maximum of 15,000) [43]. These values are used as a reference to contextualize the behavior of the material. Superglass epoxy resin (R), marketed under the brand name Pintulac, consists of two components: component A, which is the decorative resin, and component B, which is the hardener. This resin is commonly used in decorative finishes and is a liquid with moderate viscosity, amber in color, with a characteristic ammonia smell. Its properties include a boiling point of 390 °F (198 °C), 100% water solubility, a specific gravity of 0.99 g/cm^3^, and an alkaline pH. The mixture was prepared using equal proportions of components A and B. The Superglass epoxy resin used in this study corresponds to a commercial system based on bisphenol A diglycidyl ether (DGEBA), cured with an amine-type hardener, according to the information provided by the manufacturer. The exact formulation and specific nature of the additives are not detailed as they are confidential information of the manufacturer; however, this type of epoxy system is widely used in composite material applications due to its good adhesion, chemical stability, and adequate mechanical properties.

### 2.2. Methods

**Figure 1:**
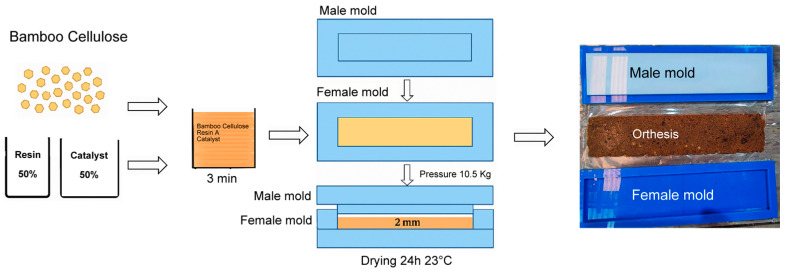
General diagram of the process for obtaining technical textiles based on bamboo cellulose.

The formation of technical textiles for the manufacture of orthoses from bamboo cellulose (BCO) begins with the use of pulverized, biodegradable bamboo material extracted using the alkaline Kraft process, which separates lignin and cellulose. The BC is mixed with the R to form BCO, as shown in the process in Figure 1.

### 2.3. Preparing the Molds

**Figure 2:**
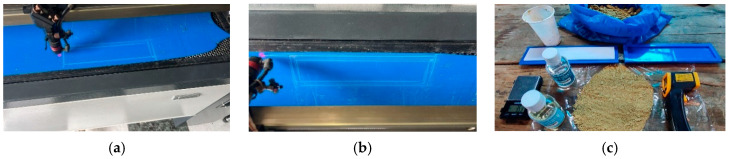
Manufacture of male and female molds from recycled acrylic using laser cutting: (**a**) laser cutting equipment, (**b**) cutting head on acrylic plate, (**c**) male and female molds obtained.

The mold manufacturing process consisted of cutting three pairs of molds using a Julong K1390 laser cutting machine manufactured in Nanjing, China with an operating voltage of 220 V and a laser power of 100 W. For this procedure, recycled flat blue acrylic with a thickness of 3 mm and white acrylic with a thickness of 2 mm were used (Figure 2a,b). The female mold has internal dimensions of 25 cm long × 5 cm wide and a depth of 6 mm. It consists of two superimposed layers, each 3 mm thick, attached to a 7 × 27 cm base plate. The male mold measures 25 cm long × 5 cm wide, with a total thickness of 4 mm, obtained by superimposing two 2 mm plates (2 × 2 = 4 mm thick). These plates are attached to a 7 × 27 cm top plate (Figure 2c).

### 2.4. Preparation of Formulations

The bamboo cellulose (BC) content range between 20% and 40% by weight was selected based on criteria of processability, structural integrity, and mechanical performance of the composite material. Contents below 20% did not provide significant reinforcement of the polymer matrix, resulting in marginal mechanical improvements and behavior dominated mainly by the resin. Conversely, contents above 40% BC caused difficulties during the process, associated with poor impregnation of the matrix and agglomeration of the reinforcement, which negatively affected the cohesion of the material. An exploratory evaluation of a 50% BC content resulted in an excessively lumpy mixture, unsuitable for compression during the formation of the BCO composite. This behavior is attributed to the total absorption of the resin by the BC, associated with the linear decrease in the glass transition temperature as the proportion of flexible epoxy resin increases [44]. This revealed structural and forming limitations, thus establishing an upper practical limit for the system investigated. On this basis, the 20–40% BC range was considered adequate to ensure a balance between effective reinforcement and processing feasibility, particularly for applications in external orthoses. Three groups of samples (S) with different formulations were established. For each compound formulation, five experimental samples were prepared according to the percentages established in Table 1.

### 2.5. Sample Manufacturing

**Figure 3:**
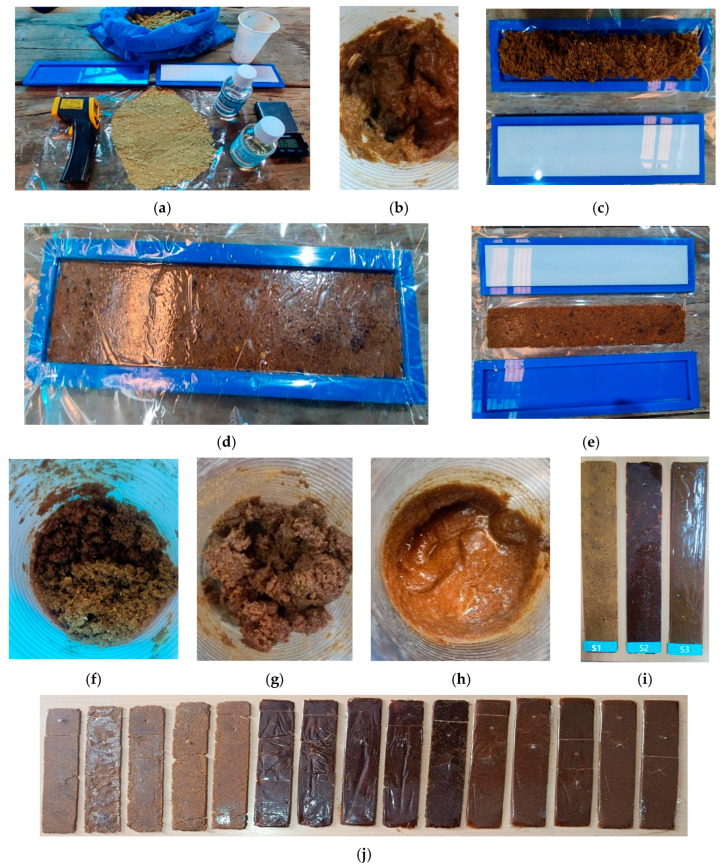
Materials, representative manufacturing stages, and final samples of the technical textile based on bamboo cellulose for applications in external orthoses and mechanical testing: (**a**) materials and equipment used; (**b**–**d**) shaping of the polymer composite in the mold; (**e**) technical textile obtained; (**f**–**h**) samples S1, S2, and S3; (**i**,**j**) final orthoses.

The materials used to obtain the S samples were made using different percentages of bamboo cellulose (BC: 40, 30, 20%) and epoxy resin (R: 60, 70, 80%), as indicated in Table 1. First, the resin (R) was weighed in a polypropylene (PP) container together with the catalyst (CT) in equal proportions (1:1 ratio), where the CT acts as an accelerating agent for the drying process at room temperature. The mixture was then stirred uniformly for 3 min at 20 °C, the temperature being measured using a Goldgood DT-500 infrared thermometer manufactured in China (Shajing City) measuring range: −50 °C to 500 °C (Figure 2c). Next, the BC and R were added to a new container and stirred manually for 5 min with a rod until a homogeneous mixture was obtained (Figure 3b). This procedure was repeated for each sample prepared in order to evaluate its reproducibility; a total of 15 samples were prepared, divided into three groups: five samples of S1, five of S2, and five of S3. The resulting mixture was placed on the female mold, which contained a 0.01 mm thick polypropylene (PPS) plastic sheet to prevent the BC polymer compound (PC) from adhering to the mold. The mixture was then spread evenly and covered with another PPS sheet (Figure 3c), and then the male mold was positioned. The sample was subjected to compression by applying a load of 10.5 kg and left to dry at room temperature (20 °C) for 24 h, obtaining a 2 mm thick laminated layer, called BCO, and considered a technical textile (Figure 3d,e). This procedure was repeated five times in each group of concentrations corresponding to S1, S2, and S3 (Figure 3f–i). It should be noted that the molding process used in this study corresponds to a laboratory methodology aimed at the initial evaluation of the material. Factors such as drying time, pressure control during forming, and homogeneous dispersion of cellulose currently limit its direct scalability. The transition to larger-scale external orthotic applications will require process optimization through more controlled forming and curing systems.

## 3. Characterization Tests on Cured Samples

**Figure 4:**
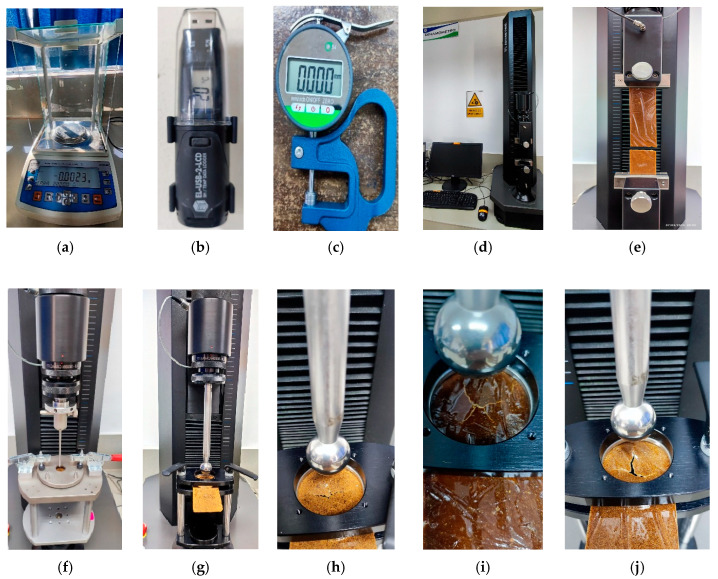
Measurement and mechanical testing equipment applied to technical bamboo cellulose textiles: (**a**–**d**) measurement and testing instruments; (**e**–**g**) configuration of tensile, puncture, and ball penetration tests; (**h**–**j**) modes of failure due to ball penetration in samples S1, S2, S3.

Characterization tests were performed on 15 samples, distributed as follows: five corresponding to group S1, five to group S2, and five to group S3. These samples were obtained and analyzed using the equipment detailed in Figure 4.

### 3.1. Tensile Strength and Elongation Resistance

The analysis to determine tensile strength and elongation was performed in accordance with ISO 9073-3, using a James Heal Titan 5 dynamometer manufactured in UK (Halifax city) with the following specifications: operating pressure of 7–10 bar (700–1000 kPa, 100–145 psi), single-phase power supply of 1.0 A, power of 60 W, dimensions of 56.8 cm depth, 40.0 cm width, and 133.9 cm height, and a minimum flow rate of 17 L/min, under ambient conditions of 20 °C and 65% relative humidity (RH). Five samples measuring 25 × 5 cm were tested for each of the concentrations S1, S2, and S3. The samples were placed between the upper and lower jaws of the Titan 5 dynamometer and subjected to a progressive load until failure (Figure 4d,e), in accordance with the mechanical conditions indicated in Table 2.

### 3.2. Puncture Resistance and Extension

Puncture resistance and extension were determined in accordance with standard EN 388, using a Titan 5 dynamometer with the following specifications: operating pressure of 7–10 bar (700–1000 kPa, 100–145 psi), single-phase power supply of 1.0 A, power of 60 W, dimensions of 56.8 cm deep, 40 cm wide, and 133.9 cm high, and a minimum flow rate of 17 L/min, under environmental conditions of 20 °C and 65% relative humidity (RH). Five 5 × 5 cm samples were tested for each of the concentrations S1, S2, and S3. The samples were placed between two flat circular plates, upper and lower, with a central hole of 1.95 cm (area: 11.94 cm^2^). The load was applied until the punch penetrated the sample (Figure 4f). The mechanical conditions used in the puncture tests are detailed in Table 3.

### 3.3. Resistance and Extension to Ball Penetration

Resistance and extension to ball penetration were determined in accordance with ASTM D3787, using a Titan 5 dynamometer with the following specifications: operating pressure of 7–10 bar (700–1000 kPa, 100–145 psi), single-phase power supply of 1.0 A, power of 60 W, dimensions of 56.8 cm depth, 40 cm width, and 133.9 cm height, and a minimum flow rate of 17 L/min, under environmental conditions of 20 °C and 65% relative humidity (RH). Five 5 × 5 cm samples were tested for each of the concentrations S1, S2, and S3. The samples were placed between two circular discs with a central hole 4.45 cm in diameter (area: 62.21 cm^2^) and subjected to progressive mechanical loading until the extension device, which has a ball at its lower end, pierced the sample (Figure 4g–j) represented in (Table 4).

### 3.4. Determination of Mass per Unit Area

The mass per unit area was determined in accordance with ISO 380. To do this, five samples measuring 25 × 5 cm were weighed using a digital scale (Figure 4a) under ambient conditions of 20 °C and 65% relative humidity (RH). Subsequently, the area of each sample was calculated in cm^2^ and extrapolated to one square meter (m^2^), obtaining the mass value of the BCO laminate, expressed in kg/m^2^. This procedure was applied to each of the 15 samples with concentrations of S1, S2, and S3.

### 3.5. Thickness Determination

The thickness of the plates was measured with a digital micrometer with an accuracy of ±0.001 mm. At five random positions under environmental conditions of 20 °C and 65% relative humidity (RH). This procedure was applied to each of the 15 samples corresponding to S1, S2, and S3 in accordance with the specifications of ASTM D1777.

## 4. Results and Analysis

### 4.1. Appearance of Samples After Curing

During the development of the BCO sample compositions, it was observed that during the process of mixing bamboo cellulose (BC) with resin (R), carried out manually with a rod for five minutes for each of the 15 samples corresponding to concentrations S1, S2, and S3, there was variability in the coloration of the samples (Figure 3f–h). This behavior is attributed to the fact that, in the S1 samples, with a composition of 40% BC and 60% R, the cellulose absorbed practically all of the resin due to its high retention capacity, which demonstrates the dominant role of cellulose in the development of the porous structure and establishes a qualitative correlation between cellulose and the porosity of the material [45]. In the S1 samples, a polymeric compound (PC) with a pasty consistency was formed, which was easily removable from the polypropylene (PP) container for placement on the female mold. When the same procedure was applied to the S2 samples (30% BC and 70% R), the PC acquired an intense brown color and a sticky texture; however, it was possible to remove it from the container without difficulty and spread it over the mold. In the case of sample S3 (20% BC and 80% R), the PC had an even more intense brown color and a pasty consistency, which made it difficult to remove and handle. After drying at 20 °C for 24 h, the BCO samples acquired different shades (Figure 3i). The S1 samples had a light yellowish color, similar to that of BC without resin (Figure 3a), and were characterized by being soft, flexible, and with a slightly shiny surface finish. The S2 samples showed a very dark brown color, greater rigidity compared to S1, and a shiny surface with no evidence of burrs. The S3 samples, on the other hand, had a brown color, considerably higher rigidity, and a very shiny surface, suggesting the migration of resin to the surface as a result of BC blooming. This phenomenon can be explained by the intrinsic properties of BC, such as its low density, hydrophobicity, chirality, and degradability [46]. The laboratory experiments were carried out under environmental conditions of 20 °C and 65% relative humidity (RH) (Figure 4b). The experimental results of the samples after curing are presented in a fusion of the parameters evaluated, where the variability observed between the results belonging to the same formulation could be attributed to several factors inherent to both the material system and the manufacturing process. In particular, localized accumulation of resin during forming, a not completely homogeneous dispersion of bamboo cellulose within the polymer matrix, and local variations in material thickness could generate differences in the final mechanical response. Likewise, slight variations in the degree of curing, associated with environmental conditions and the semi-manual nature of the process, could contribute to these fluctuations. These hypotheses are consistent with what has been reported in previous studies on polymer composite materials reinforced with natural fibers, where intra-sample variability is a common feature when highly controlled industrial processes are not used. However, the overall trend among formulations remains, suggesting that the observed variability responds mainly to procedural effects rather than to the instability of the material system indicated in Table 5.

### 4.2. Tensile Strength and Elongation at Break

**Figure 5:**
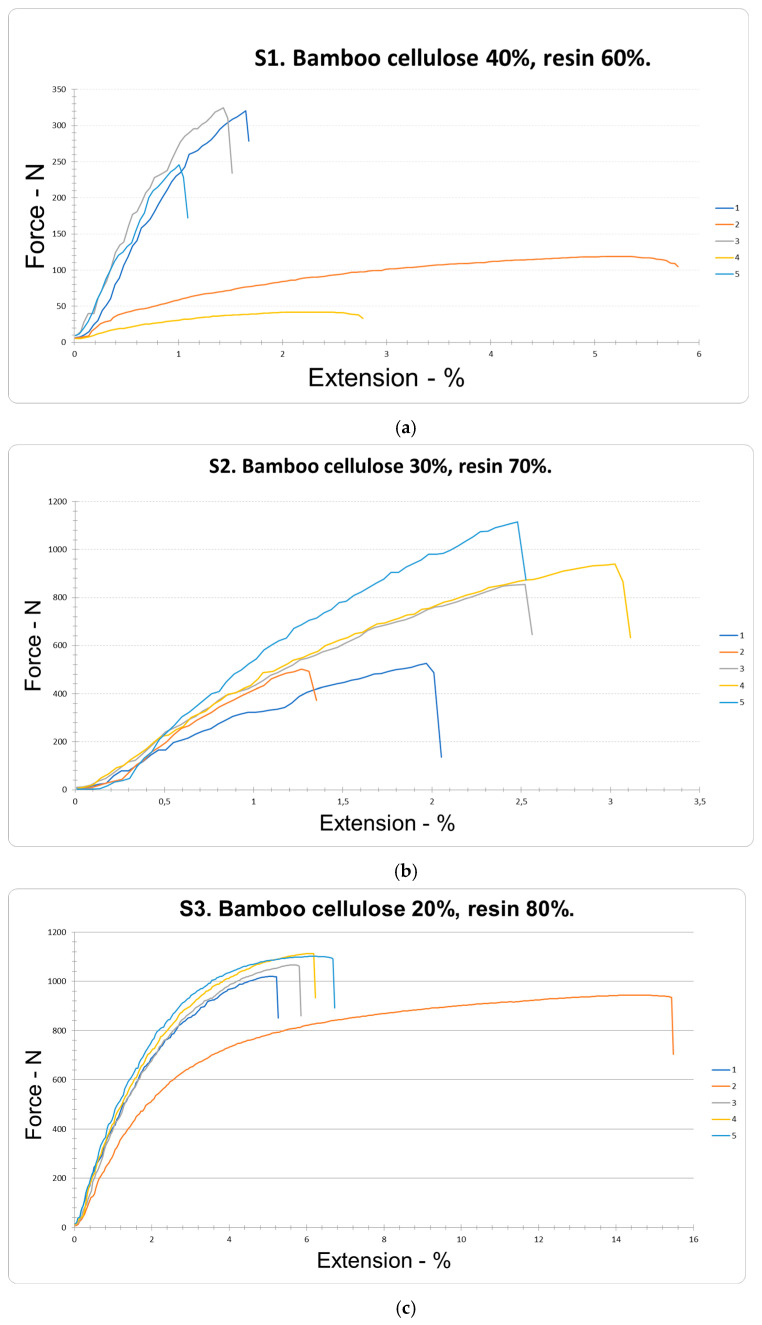
Tensile strength and elongation of samples (S1) Bamboo cellulose 40%, resin 60%; (S2) Bamboo cellulose 30%, resin 70%; and (S3) Bamboo cellulose 20%, resin 80%.

The results obtained were tabulated using Statgraphics Centurion and PAST version 4.13 software. The analysis was based on the application of a 22 factorial screening experimental design, which considered two experimental factors: bamboo cellulose (BC) and resin (R), corresponding to samples S1, S2, and S3, as well as six response variables. The experimental design had the following characteristics: number of experimental factors: 2; number of blocks: 1; number of responses: 6; number of runs: 15, including 11 central points per block; degrees of freedom for error: 11; and activated randomization. The tensile strength of the bamboo cellulose orthoses (BCO), obtained from the mixture of BC and R through the process of pressing in female and male molds, was evaluated in a total of 15 samples corresponding to S1, S2, and S3. Five independent experimental replicates were performed under the same preparation and testing conditions, following the procedures established by the standards used for mechanical characterization in order to maintain a reasonable estimate of intragroup variability and allow comparisons between formulations within the experimental scope of the study. The results were analyzed using Statgraphics Centurion and PAST 4.13 software, and the normality of the data was evaluated using the Shapiro–Wilk test. The assumption of normality was met (*p* > 0.05), with a confidence level of 95%. The differences between S1, S2, and S3 were analyzed using a one-way ANOVA, followed by a Tukey post hoc test. These indicated decisive evidence for unequal means (*p* < 0.05) in the five samples of group S1 (40% BC and 60% R) (Figure 5a). In S1, tensile strength obtained maximum values of 324.64 N and minimum values of 41.9 N, with a median of 210.45 ± 155.73 N (n = 5) and a coefficient of variation (CV) of 59.74%. These values are low compared to those reported by other authors, who reported increases from 89 to 107 MPa [47]. These samples showed high flexibility and low tensile strength. Previous studies indicate that equilibrium moisture absorption increases with relative humidity, while an increase in temperature limits this absorption [48]. By reducing the percentage of bamboo cellulose (BC) and increasing the resin content (R) in samples S2 (30% BC and 70% R) (Figure 5b), maximum values of 1115.03 N and minimum values of 502.23 N were recorded, with a median of 787.46 ± 266.59 N (n = 5) and a coefficient of variation (CV) of 33.85%. These results represent an increase of 274.17%. Consistently, another study reported the use of a similar formulation (30% bamboo fiber and 70% epoxy resin) in epoxy composites reinforced with long bamboo fiber. Extracted mechanically, compared to the bamboo cellulose used in this study, which was extracted using an alkaline chemical medium, obtaining a longitudinal elastic modulus of 8.98 GPa and a transverse elastic modulus of 2.74 GPa [49]. These results correlate directly and show that formulations with higher resin content have greater stiffness and lower deformability. The increase in resin content could contribute to a reduction in the porosity of the composite system, a lower fraction of interfiber voids, and more effective wetting of the bamboo cellulose surfaces. These effects, taken together, would promote better load transfer between the matrix and the reinforcement, which would be reflected in an increase in the overall mechanical strength of the material. The following equation was used to calculate the percentage increase:

(1) $$Percentage=\frac{Final\;value-Initial\;value}{Initial\;value}\times 100.$$

In tensile strength, by reducing the bamboo cellulose (BC) content by 10% and increasing the resin (R) proportion by 10%, the samples showed greater rigidity and a glossier surface than the S1 samples. Similarly, in the S3 samples (20% BC and 80% R) (Figure 5c) obtained maximum values of 1113.56 N and minimum values of 944.44 N, with a median of 1049.34 ± 85.57 N (n = 5) and a coefficient of variation (CV) of 6.58%. These results indicate that, as the bamboo cellulose (BC) content decreases and the resin (R) proportion increases, significant differences in tensile strength are observed compared to the S1 samples, with a statistically significant increase in the medians (Me) of the fifteen tests (*p* < 0.05). As a result, a 398.60% increase in tensile strength was obtained. This increase was calculated using the formulation with the lowest resin content evaluated in the study (S1) as a reference. Compared to previously reported studies, untreated bamboo fiber (UBF)/PP composites show an increase in tensile strength of 0.1 GO-ABF/PP composites from 31.9 MPa to 35.9 MPa (12.6% increase) [50]. These values are higher than those obtained in the present study, where a strength of 111.56 N, equivalent to 0.892 MPa, was recorded. This decrease is due to the use of untreated fiber with a length between 70 and 150 mm [43]. These results were compared with other previous studies, which compared the mechanical tensile properties of bamboo molecular hydrogel (BM-gels) with different concentrations of cellulose. A BM-gel prepared from a 7% cellulose solution by weight exhibits a tensile strength of 9.65 MPa [51]. Used for different applications, including orthotics, where polymeric compounds with higher tensile strength can be observed, compared to the bamboo cellulose used in this study, with an approximate size of 0.05 mm, it can be deduced that fiber length influences tensile strength. Furthermore, this behavior is consistent with studies that indicate that epoxy adhesives form very strong and durable bonds with most materials, which is why they are widely used in structural bonding applications, improving tensile strength [52]. However, these results differ from other studies that report smaller increases in the tensile strength of modified epoxy resins, with increases of 6.23% and 23.96%, respectively [53]. In the present study, the higher percentage increase in tensile strength suggests superior performance of the polymer composite (PC), which had a glossier surface, attributed to the increased resin content that completely coats the material, forming a more compact layer. This behavior is consistent with previous findings that report a significant improvement in the crystallinity and dimensional stability of bamboo, as well as a reduction in its porosity, hygroscopicity, and micromechanical properties [54]. On the other hand, when the proportion of bamboo cellulose (BC) increases and the resin content (R) decreases, a network of interconnected cellulose fibrils is introduced into the channels [55]. This result is reinforced by studies indicating that better resin impregnation of less dense nanocellulose networks allows maximum utilization of the stiffness and strength of cellulose nanofibrils [56]. As detailed in (Figure 5a) and summarized in (Table 6), when applying the maximums of all factors in order of priority, an optimal tensile strength (RTRAC) value of 1005.12 N was obtained from the following regression model:

TRAC resistance = −246.873 − 3.1545 × Cellulose + 20.0605 × Resin − 0.1811 × Cellulose × Resin (2)

With regard to the elongation at break of the bamboo cellulose (BC) samples obtained from the mixture of BC and resin (R) using the male and female mold pressing process, the results corresponding to the 15 samples from groups S1, S2, and S3 were evaluated. In the five S1 samples (40% BC and 60% R) (Figure 5a), a median of 2.3 ± 2.07% (n = 5) and a coefficient of variation of 72.67% were obtained, according to the data presented in Table 6. This low elongation can be attributed, according to previous reports, to the formation of a continuous pore architecture without defined orientation, generated by aggregates, which limits the elongation capacity of the material [34]. By decreasing the bamboo cellulose (BC) content by 10% and increasing the resin (R) proportion by 10% in the five samples of group S2 (30% BC and 70% R) (Figure 5b), a median of 2.25 ± 0.83% (n = 5) and a coefficient of variation of 29.55% were obtained. These results indicate low variability and a slight average decrease of 2.17% in the elongation of the polymer composite (PC). In contrast, when bamboo cellulose was reduced to 20% and resin increased to 80% in the S3 samples (20% BC and 80% R) (Figure 5c), a median of 7.55 ± 5.01% (n = 5) and a coefficient of variation of 53.58% were recorded, showing statistically significant differences between the medians (*p* < 0.05). In this case, a 228.26% increase in tensile strength was observed. This finding is consistent with studies indicating that nanofibers form highly porous systems linked by cross-linking, which allows for rapid mass transport and, at the same time, high tensile strength and elongation at break [57]. Likewise, elongation at tensile strength (ETRAC) was maximized, obtaining an optimal value of 4.86083% with the following regression model:

TRAC elongation = 2.75083 − 0.0045 × Cellulose + 0.042 × Resin − 0.000725 × Cellulose × Resin (3)

### 4.3. Resistance and Extension to Punching

**Figure 6:**
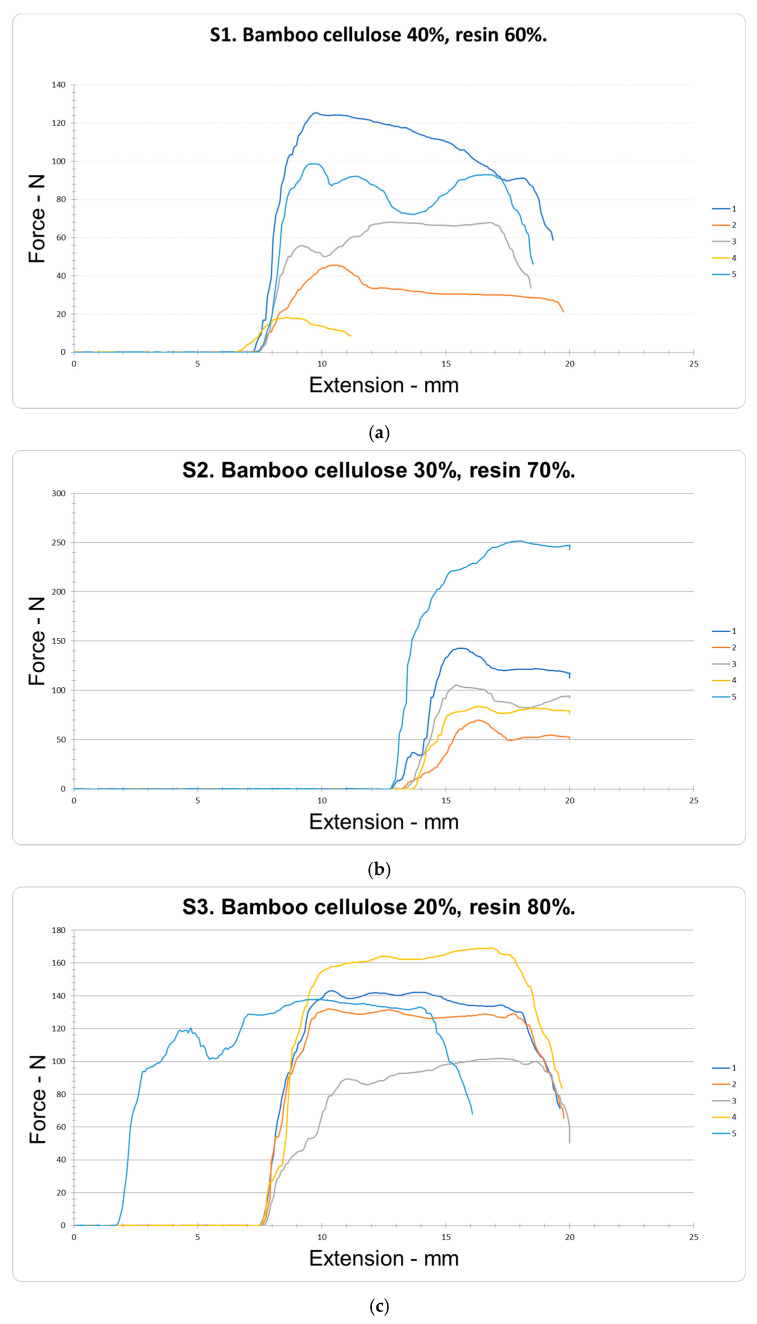
Resistance and extension to punching of the samples (S1) Bamboo cellulose 40%, resin 60%; (S2) Bamboo cellulose 30%, resin 70%; and (S3) Bamboo cellulose 20%, resin 80%.

The puncture resistance of the bamboo cellulose orthosis (BCO), obtained from a mixture of bamboo cellulose (BC) and resin (R) using a male and female mold pressing process, showed the following results for samples S1, S2, and S3. In the five samples of group S1 (40% BC and 60% R) (Figure 6a), maximum values of 125.24 N and minimum values of 18.05 N were recorded, with a median of 100.0 N. In the five samples of group S2 (60% BC and 40% R) (Figure 6b), maximum values of 125.24 N and minimum values of 18.05 N were recorded, with a median of 100.0 N. In the five samples of group S3 (60% BC and 40% R) (Figure 6c), maximum values of 125.24 N and minimum values of 18.05 N were recorded, with a median of 100.0 N. In group S1 (40% BC and 60% R) (Figure 6a), maximum values of 125.24 N and minimum values of 18.05 N were recorded, with a median of 71.14 ± 52.49 N (n = 5) and a coefficient of variation (CV) of 59.52% (Table 7). These results show low puncture resistance. By decreasing the bamboo cellulose (BC) content and increasing the resin (R) in samples S2 (30% BC and 70% R) (Figure 6b), maximum values of 251.64 N and minimum values of 69.96 N were found, with a median of 130.68 ± 90.47 N (n = 5) and a coefficient of variation (CV) of 55.84%. These values represent an 83.69% increase in puncture resistance. In the case of samples S3 (BC 40%, R 80%) (Figure 6c), maximum values of 169.03 N and minimum values of 101.76 N were recorded, with a median of 24.12 ± 29.91 N (n = 5) and a coefficient of variation (CV) of 17.64%. These results indicate that, by reducing the proportion of bamboo cellulose (BC) and increasing the resin (R), the puncture resistance increases significantly compared to the S1 samples, with statistically significant differences between the medians (Me) of the fifteen tests (*p* < 0.05). Likewise, most of the values were concentrated in the P75 percentile (142.59 N), achieving an overall increase of 92.25% in puncture resistance. Similarly, when analyzing the values corresponding to the different proportions of BC and R, punch resistance maximization (RPUNZ) was prioritized, obtaining an optimal value of 151.23 N from the following regression model:

PUNZ resistance = 330.941 − 3.4295 × Cellulose − 2.2645 × Resin + 0.020625 × Cellulose × Resin (4)

In the extension tests performed on the bamboo cellulose (BC) samples mixed with resin (R), obtained through the male and female mold pressing process, the results corresponding to the 15 samples from groups S1, S2, and S3 were evaluated. In the five samples from group S1 (40% BC and 60% R) (Figure 6a), a median of 10.22 ± 1.87 mm (n = 5) and a coefficient of variation of 14.76% were obtained, according to the data presented in Table 7. By reducing the bamboo cellulose (BC) content and increasing the resin (R) proportion in the S2 samples (30% BC and 70% R) (Figure 6b), a median of 16.33 ± 1.25 mm (n = 5) and a coefficient of variation of 6.20% were recorded. These results show an increase of 6.11 mm in extension, with most values falling within the P75 percentile (16.33 mm), corresponding to an average increase of 59.78% in the extension of the polymeric compound (PC). This behavior can be attributed to adequate interfacial adhesion between the epoxy resin and the cellulose fibers, favored by interaction through hydrogen bonds [58]. In contrast, by reducing the bamboo cellulose content to 20% and increasing the resin to 80% in the S3 samples (Figure 6c), a median of 12.37 ± 4.64 mm (n = 5) and a coefficient of variation of 29.05% were obtained, with statistically significant differences between the medians (*p* < 0.05). In this case, the increase was 2.65 mm, equivalent to a 25.92% increase in extension. These results indicate that, at higher resin (R) contents, the elongation of the material tends to decrease in the S3 samples. Considering the different concentrations of BC and R, the maximum extension in the punch test (PUNZ) was achieved with an optimal value of 15.2008 mm, which fits the following regression model:

PUNZ extension = 42.1408 − 1.3405 × Cellulose − 0.3595 × Resin + 0.017325 × Cellulose × Resin (5)

### 4.4. Analysis of Resistance and Extension to Ball Penetration

**Figure 7:**
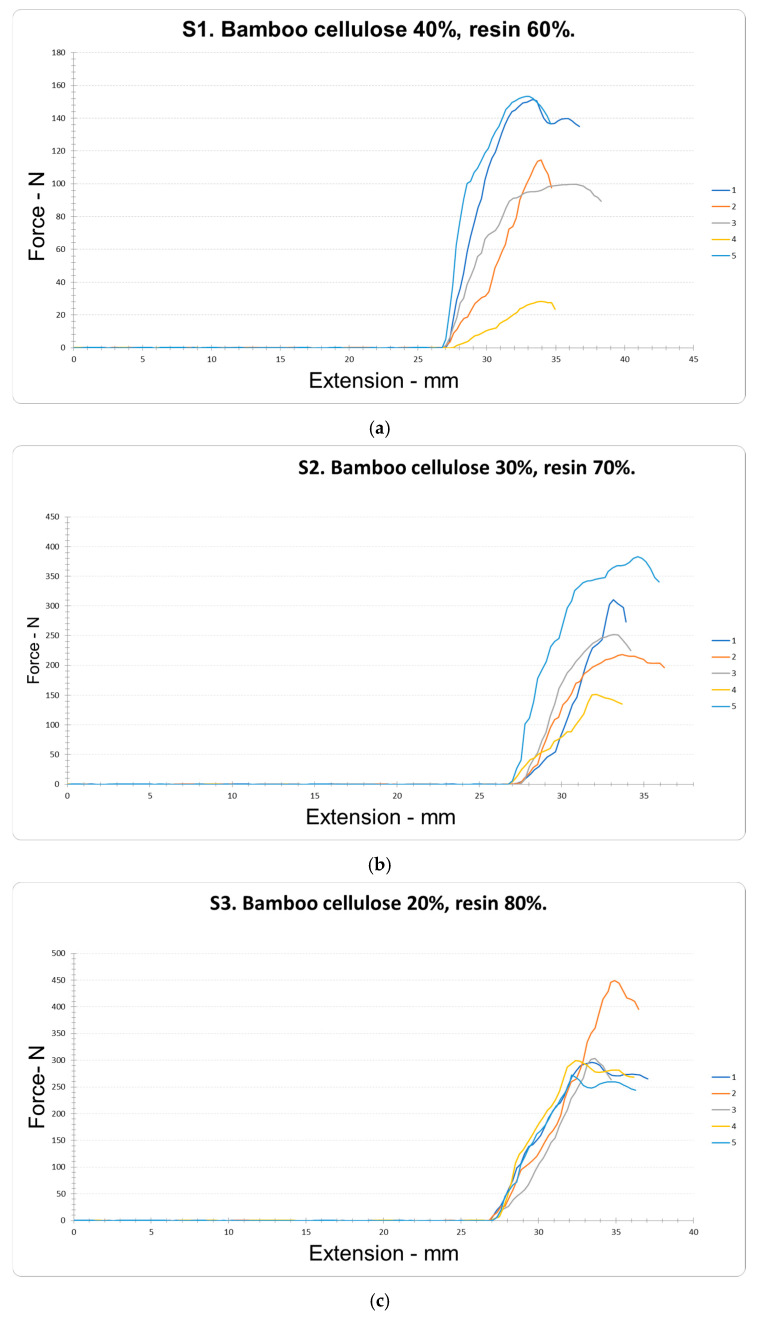
Resistance and elongation to ball penetration in five tests (**a**–**c**) for samples (S1) Bamboo cellulose 40%, resin 60%; (S2) Bamboo cellulose 30%, resin 70%; and (S3) Bamboo cellulose 20%, resin 80%.

For the ball penetration resistance test, five samples from group S1 (40% BC and 60% R) were evaluated (Figure 7a). In this case, maximum values of 153.33 N and minimum values of 28.26 N were recorded, with a median of 109.44 ± 63.21 N (n = 5) and a coefficient of variation (CV) of 46.59%. These results indicate low ball penetration resistance. In contrast, by reducing the bamboo cellulose (BC) content and increasing the resin (R) proportion in samples S2 (30% BC and 70% R) (Figure 7b), maximum values of 383.35 N and minimum values of 151.23 N were obtained with a median of 262.94 ± 109.85 N (n = 5) and a coefficient of variation (CV) of 33.70%. These results represent a 140.25% increase in ball penetration resistance compared to sample S1. As the bamboo cellulose content continued to decrease and the resin content increased in samples S3 (20% BC and 80% R) (Figure 7c). Maximum values of 449.09 N and minimum values of 272.24 N were recorded, with a median of 323.98 ± 1.39 N (n = 5) and a coefficient of variation (CV) of 3.37%. These results show that reducing the proportion of BC and increasing the R resin content produces significant differences in ball penetration resistance compared to the S1 samples, with a 196.03% increase in the medians (Me) of the fifteen tests (*p* < 0.05). In this context, the conformation of the polymeric compound (PC) to obtain the BCO material shows a clear trend towards increased ball penetration resistance (BPR). These results are consistent with previous studies reporting that the addition of between 18 and 23% by weight of cellulose nanofibrils (CNF) in epoxy matrices significantly increases the modulus, strength, and deformation of the resulting composites [59]. The changes observed are illustrated in Figure 7c and summarized in Table 8. By maximizing ball penetration resistance (RPEBOL), the best value of 309.339 N was achieved, characterized by the regression model with the following equation:

PEBOL resistance = 1013.91 − 26.3875 × Cellulose − 9.122 × Resin + 0.30875 × Cellulose × Resin (6)

In the ball penetration extension test, five samples from group S1 (40% BC and 60% R) were evaluated (Figure 7a). Maximum values of 36.25 mm and minimum values of 32.84 mm were recorded, with a median of 34.07 ± 63.21 mm (n = 5) and a coefficient of variation (CV) of 3.83%. These results indicate a high ball penetration extension, in accordance with data reported by other authors, who attribute this behavior to the strong interactions between the cellulose fibers and the epoxy network and the creation of a percolation network joined by hydrogen bonds between cellulose fibers [60]. By reducing the bamboo cellulose (BC) content and increasing the resin (R) proportion in samples S2 (30% BC and 70% R) (Figure 7b), maximum values of 34.62 mm and minimum values of 32.12 mm were obtained, with a median of 33.35 ± 1.13 mm (n = 5) and a coefficient of variation (CV) of 2.73%. These results show a slight decrease of 2.11% in ball penetration extension. Similarly, by continuing to decrease the bamboo cellulose content to 20% and increasing the resin to 80% in samples S3 (Figure 7c), maximum values of 34.93 mm and minimum values of 32.13 mm were recorded, with a median of 33.31 ± 1.39 mm (n = 5) and a coefficient of variation (CV) of 3.37%. These results indicate that, by reducing the proportion of BC and increasing the R content, there are no statistically significant differences in ball penetration extension compared to the S1 samples, according to the medians (Me) of the fifteen tests (*p* > 0.05). Overall, the findings reflect a slight decrease of 2.23% in ball penetration extension. In this context, the conformation of the polymeric compound (PC) for obtaining the BCO material shows a slight tendency to decrease ball penetration extension (BPE). This behavior can be attributed to the fact that, at high loads, the particles tend to agglomerate due to strong van der Waals forces [61]. As detailed in Figure 7c and summarized in Table 8, no significant differences in ball penetration extension were observed between the samples (*p* > 0.05). Consequently, an optimal EPEBOL value of 34.054 mm was obtained, described by the following regression equation:

PE BOL extension = 36.314 − 0.1415 × Cellulose − 0.0415 × Resin + 0.0021 × Cellulose × Resin (7)

### 4.5. Correlation Between Samples

**Figure 8:**
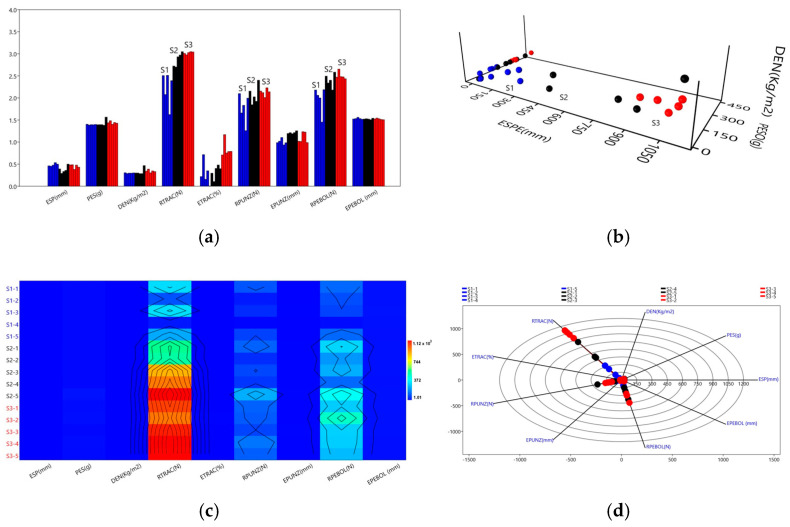
Graphical representation of experimental factors and response variables using estimated surfaces, correlation matrix, and radar diagram. (**a**) Comparison of RTRAC and ETRAC between S1, S2, and S3. (**b**) PES-ESP-DEN relationship using estimated surface area. (**c**) Correlation matrix of experimental variables. (**d**) Radar diagram of experimental factors and response variables.

In Figure 8a,b, corresponding to the bar and surface graphs, it can be observed that samples S1, S2, and S3 have uniform weight averages (PES), with an overall mean of 26.40 ± 0.85 g and a coefficient of variation (CV) of 12.53%, with no statistically significant differences (*p* > 0.05). This behavior indicates that the percentages of resin (R) and bamboo cellulose (BC) used do not generate a significant increase in the total weight of the orthoses, which is attributed to the density of the resin used (0.99 g/cm^3^). In terms of thickness (TH), no statistically significant differences were observed (*p* > 0.05), with a mean value of 2.76 ± 0.1 mm and a CV of 15.37%. This uniformity is due to the 10.5 kg compression load applied to the male mold during the forming process, which allowed homogeneous surfaces to be obtained in the bamboo cellulose orthosis (BCO) samples. However, slight variations were identified in the S2 samples. Density (DEN) showed statistically significant differences (*p* < 0.05), with a mean of 2.10 ± 0.06 kg/m^2^ and a CV of 12.64%. An outlier was identified in sample S2 (Figure 8b), while fluctuations attributable to density variability were observed in samples S1 and S3. This behavior suggests that a higher resin content (R) in the pressed polymeric compound (PC) favors the formation of a more porous surface, which hinders a completely uniform distribution of the material over the female mold during the shaping of the BCOs. In Figure 8d, corresponding to the radar chart that integrates the experimental factors and response variables, analysis using ANOVA and Kruskal–Wallis showed statistically significant differences between the means of groups S1, S2, and S3 (*p* < 0.05; F = 35.15). Considering the percentage of participation, it was estimated that approximately 65% of the total variability of the data is associated with the effect of the omega treatment (ω2 = 0.65), which confirms a significant effect. The variables with the greatest influence were tensile strength (RTRAC) with an average of 682.41 N; ball penetration resistance (RPEBOL) with 232.11 N; and puncture resistance (RPUNZ) with 112.85 N. In contrast, the variables related to extension had the following averages: ball penetration extension (BPE), 33.57 mm; punch extension (PEX), 13.14 mm; tensile strength elongation (TSEL), 4.60 mm (4.03%). These results indicate that as extension increases, the resistance of the bamboo cellulose orthosis (BCO). The correlations observed between thickness, weight, and density highlight the influence of geometric control on the mechanical response of the material. The variations in thickness recorded, associated with the manual nature of the molding process, may introduce slight heterogeneities; however, the overall trends remain consistent between formulations, supporting the internal reproducibility of the method under the experimental conditions evaluated. In Figure 8d; that is, when resistance is greater, extension also increases, evidencing a directly proportional relationship between the two variables. Figure 8c illustrates the experimental factors and correlated response variables, represented by a color scale ranging from light blue (low correlation) to red (high correlation) for S1, S2, and S3. This visualization shows that the variables thickness (ESP), weight (PES), and density (DEN) are strongly related to each other (*p* > 0.05), which is attributed to the standardized use of male and female molds, with a constant thickness of 2 mm and a uniform pressure of 10.5 kg applied to the 15 S samples (Figure 2c). The mechanical behavior observed suggests the presence of mixed failure modes, where fracture of the polymer matrix coexists with mechanisms of extraction or slippage of the cellulose reinforcement. These mechanisms are consistent with the expected behavior in natural fiber-reinforced composites and support the contribution of the cellulose reinforcement to the mechanical response of the material. On the other hand, tensile strength (RTRAC) showed statistically significant differences (*p* < 0.05), demonstrating that the S3 samples achieved higher values compared to the S1 and S2 samples. This increase is associated with the increase in resin content (R) from 60% to 80% and the reduction in bamboo cellulose (BC) from 40% to 20%. In contrast, the variables of puncture resistance (PR) and ball penetration resistance (BPR) did not show statistically significant differences (*p* > 0.05) (Figure 8c), although a slight increase in their resistance was observed. Regarding the elongation and extension variables, both tensile elongation (ETRAC) and puncture extension (EPUNZ) showed significantly different effects (*p* < 0.05) (Figure 8a), with average values of 4.03 mm and 13.14 mm, respectively. In contrast, ball penetration extension (EPEBOL) showed no significant differences, remaining at a constant average value of 33.57 mm (*p* > 0.05). The joint comparison of mechanical responses suggests that the improvements observed in tensile strength, particularly in formulation S3, are not proportionally reflected in the properties associated with punching and ball penetration. This divergence could be related to changes in the damage mechanisms of the material, where greater stiffness and efficiency in load transfer favor the response under uniaxial loads, while under localized stresses, deformation and crack propagation play a more dominant role according to the (Figure 8a).

Finally, this study focuses on the initial mechanical characterization of the technical textile developed. In this context, it is acknowledged that complementary evaluations, such as biocompatibility, moisture absorption, accelerated aging, microstructural analysis, and durability under cyclic load tests, were not addressed at this stage due to limitations in specialized equipment. These evaluations are essential steps for future validation before eventual clinical application in external orthoses.

## 5. Conclusions

This study demonstrates the relevance of bamboo cellulose (BC) as a natural and sustainable raw material for use in bamboo cellulose-based orthoses (BCO), in combination results with epoxy resin (R), for the development of technical textiles for medical applications. The indicate that, when preparing polymer composites (PC) with concentrations of 20, 30, and 40% bamboo cellulose (BC), combined with 80, 70, and 60% epoxy resin (R), respectively, in the 15 samples corresponding to groups S1, S2, and S3, representative averages with greater influence in the strength and elongation tests were obtained. In terms of tensile strength (RTRAC), the most influential samples were S3 (20% BC and 80% R) with a value of 1049.34 ± 85.57 N (n = 5), representing an increase of 398.60% compared to group S1. Regarding tensile elongation (ETRAC), the best results were recorded in samples S3, with a median of 7.55 ± 5.01% (n = 5) and an increase of 228%. In relation to puncture resistance (RPUNZ), the S3 samples had the highest values, with 24.12 ± 29.91 N (n = 5), corresponding to an increase of 92.25%. The greatest punch extension (EPUNZ) was obtained in the S2 samples (30% BC and 70% R), with a value of 16.33 ± 1.25 mm (n = 5), equivalent to an increase of 59.78%. In terms of ball penetration resistance (RPEBOL), the best.

## Figures and Tables

**Table 1:** Composition of formulations in percentages of bamboo cellulose and resin.

Sample (S)	Bamboo Cellulose (BC) (%)	Resin (R) (%)
S1	40	60
S2	30	70
S3	20	80

**Table 2:** Mechanical conditions used to determine tensile strength and elongation according to ISO 9073-3.

Test Details	
Test Name:	Resistance sample
Test tubes:	5
Required addresses:	MD
Gag Plan:	T27
Jaw separation:	200.00 mm
Force Control Gain:	25
Load cell:	5000 N
Load Cell SN:	SERIAL NUMBER
Version:	5.0.10.0
Firmware:	V2.7
Titan SN:	Not Licensed
Tested by:	Administrator
Procedure configuration	
Breakage detection:	10%
Claim:	5.00 N
Speed:	100.00 mm/min

**Table 3:** Mechanical conditions used to determine resistance and extension to puncturing, according to standard EN 388.

Test Details	
Required addresses:	Punching
Test tubes:	5
Required addresses:	Not applicable
Gag Plan:	T32
Jaw Separation(s):	0.00
Force Control Gain:	25
Load cell:	5000 N
Version:	SERIAL NUMBER
Firmware:	5.0.10.0
Tested by:	V2.7
Titan SN:	Not Licensed
Tested by:	Administrator
Procedure configuration	
Breakage detection:	50%
Claim:	20.00 N
Speed:	100.00 mm/min

**Table 4:** Mechanical conditions used to determine resistance and ball indentation according to ASTM D3787.

Test Details	
Test Name:	Ball penetration
Test tubes:	5
Required addresses:	Not applicable
Gag Plan:	T20A
Force Control Gain:	25
Load Cell SN:	SERIAL NUMBER
Version:	5.0.10.0
Firmware:	V2.7
Titan SN:	Not Licensed
Tested by:	Administrator
Procedure configuration	
Breakage detection:	10%
Claim:	There is no claim (disconnected claim)
Speed:	305.00 mm/min

**Table 5:** Experimental results and response factors of samples with different concentrations of bamboo cellulose (BC) and resin.

Sample (S)	Thickness (mm)	Weight (g)	Density (Kg/m^2^)	Tensile Strength (N)	Elongation at Tensile Strength (%)	Punching Resistance (N)	Extension in the Punching (mm)	Ball Penetration Resistance (N)	Extension in Ball Penetration (mm)
S1-1	2.89	25.27	2.02	320.77	1.65	125.24	9.72	151.43	33.35
S1-2	2.83	24.27	1.94	119.04	5.19	45.57	10.47	114.42	33.94
S1-3	3.00	24.79	1.98	324.64	1.43	68.07	12.66	99.77	36.25
S1-4	3.42	24.61	1.97	41.90	2.23	18.05	8.64	28.26	33.94
S1-5	3.15	25.04	2.00	245.90	1.01	98.75	9.63	153.33	32.84
S2-1	2.46	24.61	1.99	526.31	1.97	142.59	15.65	310.58	33.15
S2-2	1.95	24.83	1.99	502.23	1.27	69.96	16.30	217.89	33.69
S2-3	2.15	24.73	1.92	855.08	2.52	105.55	15.39	251.64	33.16
S2-4	2.28	23.97	1.92	938.65	3.02	83.67	16.33	151.23	32.12
S2-5	3.16	36.65	2.93	1115.03	2.48	251.64	17.99	383.35	34.62
S3-1	3.03	27.31	2.19	1019.90	5.10	143.04	10.40	296.3	33.46
S3-2	3.06	30.43	2.43	944.44	14.74	132.12	10.32	449.09	34.93
S3-3	2.40	25.14	2.01	1067.02	5.65	101.76	17.15	303.20	33.66
S3-4	3.00	27.69	2.22	1113.56	6.10	169.03	16.77	299.06	32.37
S3-5	2.70	26.70	2.14	1101.78	6.14	137.76	9.73	272.24	32.13

**Table 6:** Maximum strength and elongation results for samples with different concentrations of bamboo cellulose and resin.

MD Results							
	S1 (BC 40%, R 60%)			S2 (BC 30%, R 70%)		S3 (BC 20%, R 80%)	
Test Specimen	Maximum Force (N)	Elongation (%)	Extension (mm)	Maximum Force (N)	Elongation (%)	Maximum Force (N)	Elongation (%)
1	320.77	1.65	3.3	526.31	1.97	1019.9	5.1
2	119.04	5.19	10.38	502.23	1.27	944.44	14.74
3	324.64	1.43	2.86	855.08	2.52	1067.02	5.65
4	41.9	2.23	4.46	938.65	3.02	1113.56	6.1
5	245.9	1.01	2.02	1115.03	2.48	1101.78	6.14
Mean	210.45	2.3	4.6	787.46	2.25	1049.34	7.55
Standard deviation	125.73	1.67	-	266.59	0.6655	69.03	4.04
Confidence limits	±155.87	±2.07	-	±330.49	±0.8250	±85.57	±5.01
Coefficient of variation	59.74%	72.67%	-	33.85%	29.55%	6.58%	53.58%

**Table 7:** Resistance and extension to punching of samples with different concentrations of bamboo cellulose and resin.

	S1 (BC 40%, R 60%)		S2 (BC 30%, R 70%)		S3 (BC 20%, R 80%)	
Test Specimen	Puncture Resistance (N)	Extension (mm)	Puncture Resistance (N)	Extension (mm)	Puncture Resistance (N)	Extension (mm)
1	125.24	9.72	142.59	15.65	143.04	10.4
2	45.57	10.47	69.96	16.3	132.12	10.32
3	68.07	12.66	105.55	15.39	101.76	17.15
4	18.05	8.64	83.67	16.33	169.03	16.77
5	98.75	9.63	251.64	17.99	137.76	9.73
Mean	71.14	10.22	130.68	16.33	136.74	12.87
Standard deviation	42.34	1.51	72.97	1.01	24.12	3.74
Confidence limits	±52.49	±1.87	±90.47	±1.25	±29.91	±4.64
Coefficient of variation	59.52%	14.76%	55.84%	6.20%	17.64%	29.05%

**Table 8:** Resistance and ball penetration extension of samples with different concentrations of bamboo cellulose and resin.

	S1 (BC 40%, R 60%)		S2 (BC 30%, R 70%)		S3 (BC 20%, R 80%)	
Test Specimen	Maximum Force (N)	Extension (mm)	Maximum Force (N)	Extension (mm)	Maximum Force (N)	Extension (mm)
1	151.43	33.35	310.58	33.15	296.3	33.46
2	114.42	33.94	217.89	33.69	449.09	34.93
3	99.77	36.25	251.64	33.16	303.2	33.66
4	28.26	33.94	151.23	32.12	299.06	32.37
5	153.33	32.84	383.35	34.62	272.24	32.13
Mean	109.44	34.07	262.94	33.35	323.98	33.31
Standard deviation	50.98	1.31	88.61	0.9111	70.97	1.12
Confidence limits	±63.21	±1.61	±109.85	±1.13	±87.98	±1.39
Coefficient of variation	46.59%	3.83%	33.70%	2.73%	21.91%	3.37%

## Data Availability

The original contributions presented in this study are included in the article. Further inquiries can be directed to the corresponding author.
